# Multiple-Criteria Decision-Making (MCDM) techniques to study the behavior of dendrimers using topological indices

**DOI:** 10.1371/journal.pone.0294515

**Published:** 2023-11-30

**Authors:** Xuewu Zuo, Maryam Akhtar, Adnan Aslam, Ferdous M. Tawfiq, Salma Kanwal

**Affiliations:** 1 General Education Department, Anhui Xinhua University, Hefei, China; 2 Department of Mathematics, Lahore College for Women University, Lahore, Pakistan; 3 Department of Natural Sciences and Humanities, University of Engineering and Technology, Lahore(RCET), Pakistan; 4 Mathematics Department, College of Science, King Saud University, Riyadh, Saudi Arabia; King Khalid University, SAUDI ARABIA

## Abstract

Topological indices provide a mathematical language for capturing molecular structure, symmetry, and predicting properties. Dendrimers are microscopic bilaterally symmetrical molecules with a well-defined homogeneous nanoparticles structure, often consisting of a symmetric center, inner shell, and outer shell. In this work, first we compute some degree-based topological indices of Porphyrin (*D*_*n*_*P*_*n*_),Poly (Propyl) Ether Imine(*PETIM*), Zinc porphyrin (*DPZ*_*n*_), and Polyamidoamine (*PAMAM*) dendrimers. Then, we use multi-criteria decision making (MCDM) techniques to establish the weighted evaluation of dendrimer classes based on certain topological indices. For weighted analysis we correlate the properties of benzene derivatives with topological invariants. Finally, based on the multi-criteria decision making techniques namely TOPSIS, SAW and MOORA method, we have ranked the dendrimer structures based on their properties.

## Section 1: Introduction

Chemical graph theory is a branch of mathematics and theoretical chemistry that focus on the study of molecular structure, bonding, and properties using graph theory tools. In this field, a molecule is represented by a graph where the atoms represent the vertices of a graph and the edges represent the bonds between the vertices. A graph *G* = *G*(*V*, *E*) having a vertex set *V*(*G*) and an edge set *E*(*G*) is called connected if any pair of vertices in *G* is connected by a path. The distance between two vertices g and h is the length of the shortest path between them andis denoted by *d*(*g*, *h*). The degree of a vertex *u* is denoted by *d*_*u*_ and is the number of edges incident to it. For basic concepts related to graph theory see [1].

Topological index is a real number assigned to a molecular graph and is used to predict its physical and chemical properties. Weiner index is one of the first indices introduced by Harold Wiener in 1947 [2], while he was working on the boiling point of paraffin. Since then, over 3000 topological indices have been recorded in Chemical Data Bases. Mathematicians and chemists are both interested in this field of inquiry. Topological graph indices are being investigated all around the world, since there is a great deal of interest in this topic. To identify the chemical properties of developing nanotubes and nanomaterials, a large number of chemical tests are required. Chemical investigations show that out of all, no individual topological index is strong enough to predict numerous physic-chemical characteristics, when used collectively; these topological indices can do so to some extent.

(1)In [3], A.Subhashini and J.BaskarBabujee introduced reduced forgotten topological index as

$$RF(G)=\sum_{gh∊E(G)}\left[{\left(d(g)-1\right)}^{2}+{\left(d(h)-1\right)}^{2}\right].$$ (1)

(2)Randic et al. [4] introduced the Randi index *R*(*G*). It was defined as

$$R(G)=\sum_{gh∊E(G)}\frac{1}{\sqrt{d_{g}d_{h}}}.$$ (2)

(3)The Reciprocal Randic index *RR*(*G*) was described as

$$RR(G)=\sum_{gh∊E(G)}\sqrt{d_{g}d_{h}}.$$ (3)

(4) The Harmonic index *H*(*G*) was introduced by Zhong [5] and is defined as

$$H(G)=\sum_{gh∊E(G)}\frac{2}{d(g)+d(h)}.$$ (4)

For the results related to the applications of the topological indices and their computation for different molecular structures see [6–10].

MCDM means the process of determining the best feasible solution according to established criteria and problems that are common occurrences in everyday life. There are several criteria to consider. Making decisions in the face of several characteristics that are in contradiction is referred to as multi-criteria decision-making (MCDM) [11]. An integrated Analytic Hierarchy Process–Treatment of Differential Equations technique for administration of the supply chain [12], assessing bridge risk [13], material choice [14], and evaluation of weapons system [15], assembling environments [16], are all examples of MCDM in action. In multi-criteria decision-making challenges, a decision maker (DM) must select the most suitable option from a group of candidate solutions that meets the assessment criteria. The DM’s ability to make a scientific judgment by balancing these competing traits is mostly dependent on his or her experience. Many MCDM techniques are invented to solve these obstacles. Multiple incommensurable and contradictory criteria, different plans and measures among the criteria, and the existence of dissimilar options are all prevalent aspects of MCDM problems [17]. MCDM is involved in constructing and solving multi-criteria decision and planning problems [18].

The paper is structures as follows: In section 2, first we compute the above defined topological indices of Porphyrin, Poly (Propyl) Ether Imine, Zinc Porphyrin and Polyamidoamine dendrimers. We find the correlation of these topological indices with some properties of benzene derivatives in section 3. In section 4, we use these correlation coefficients to calculate the weights in the multi-criteria decision making techniques to rank these dendrimers depending on their properties. The calculation of multicriteria decision making are presented in section 5.

## Section 2: Topological indices of dendrimer structures

Vögtle created the first dendrimers in 1978 using divergent synthesis processes [19]. Dendrimers are highly branched macromolecules with a well-defined and precise three-dimensional structure, resembling a tree-like architecture. The term "dendrimer" is derived from the Greek word "dendron," meaning tree. The key components of a dendrimer structure include a central core, branches, and terminal functional groups. The growth of branches occurs through a series of repeated steps or generations. Dendrimers have several uses in nanoscience and medicine. Dendrimers are one of the few polymers whose shape and architecture can be carefully regulated. Dendritic macromolecules have unique features due to their well-defined structure, making them ideal for a variety of applications, particularly in the biomedical industry. Dendrimers are a cutting-edge delivery technology for anticancer medications with increased solubility and reduced toxicity, as well as for targeted administration to cancerous cells. *PPI*, *PAMAM*, *and PLL dendrimers*, have been primarily investigated for anticancer drug delivery. Dendrimers are used for delivery of drugs in two ways:

- formulation
- Nano construct.

Medications are physically entrapped in a dendrimer utilizing non-covalent contacts in the formulation strategy, whereas drugs are covalently attached to dendrimers in the nano construct approach.

In this section, we calculate some degree-based topological indices of four common dendrimer classes i.e., Porphyrin(*D*_*n*_*P*_*n*_),Poly (Propyl) Ether Imine (*PETIM*), Zinc Porphyrin(*DPZ*_*n*_) and Polyamidoamine(*PAMAM*) dendrimers.

### 2.1 Porphyrin dendrimer (D_n_P_n_)

**Fig 1:**
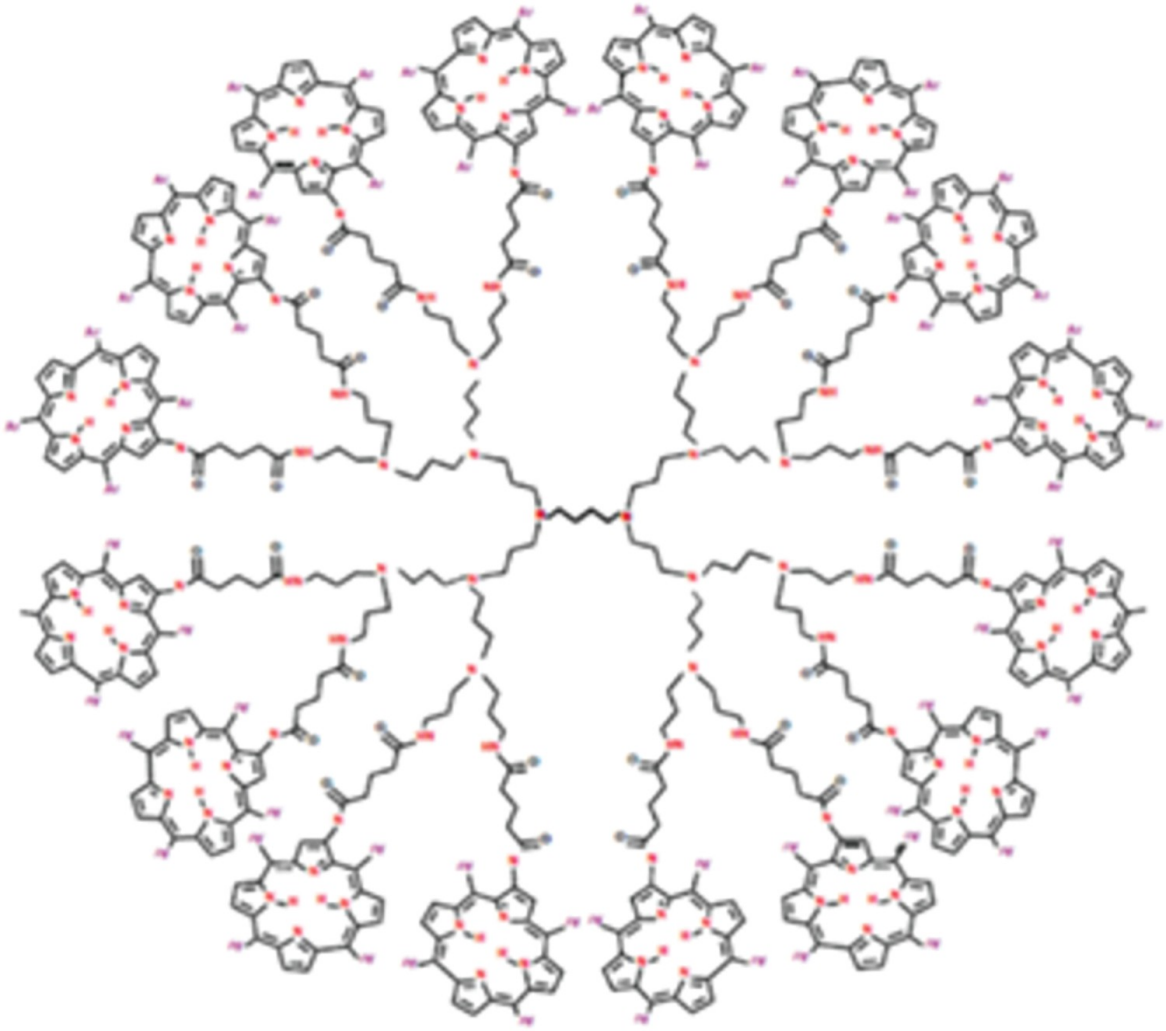
Porphyrin dendrimer *D*_*n*_*P*_*n*_.

Porphyrin dendrimers are non-invasive chemicals that contain large heterocyclic-aromatic units within the dendritic architecture and display remarkable photodynamic characteristics. In porphyrin dendrimer structure the central core is composed of porphyrin units. The porphyrin units are typically used as building blocks for dendritic architecture, creating and highly branched dendrimeric structure with porphyrin containing nodes. The molecular graph of porphyrin dendrimer with *n* growth stages is depicted in Fig 1.Observe that *n* = 2*m*, where *m* ≥ 2.The molecular graph of *D*_*n*_*P*_*n*_ comprises of four identical branches and a central core with five extra edges. It is easy to calculate that *D*_*n*_*P*_*n*_ has a total of 96*n* − 10 vertices in which26*n* vertices are of degree 1, 34*n* − 8 vertices are of degree 2, 28*n* − 2 vertices are of degree 3, and the remaining 8n vertices are of degree 4. The total number of edges of *D*_*n*_*P*_*n*_ are 105*n* − 11. Let *E*_*ij*_ denote the number of edges of *D*_*n*_*P*_*n*_ whose end vertices have degree *i* and *j* respectively. Then the edge partition of *D*_*n*_*P*_*n*_ based on the degree of the end vertices of each edge is as follows: $E_{13}=2n,E_{14}=24n,E_{22}=10n-5,E_{23}=48n-6,E_{33}=13n$, and *E*_34_ = 8*n*. Now, using these values in the definitions of the reduced Forgotten index, the Randic index, the reduced Randic index and the Harmonic index we get the following expressions.

- $RF(D_{n}P_{n})=692n-40$.
- $R(D_{n}P_{n})=44.39335289n-4.949489743$.
- $RR(D_{n}P_{n})=255.7524222n-24.69693846$.
- $H(D_{n}P_{n})=41.41904762n-4.9$.

### 2.2 Zinc porphyrin dendrimer (DPZ_n_)

**Fig 2:**
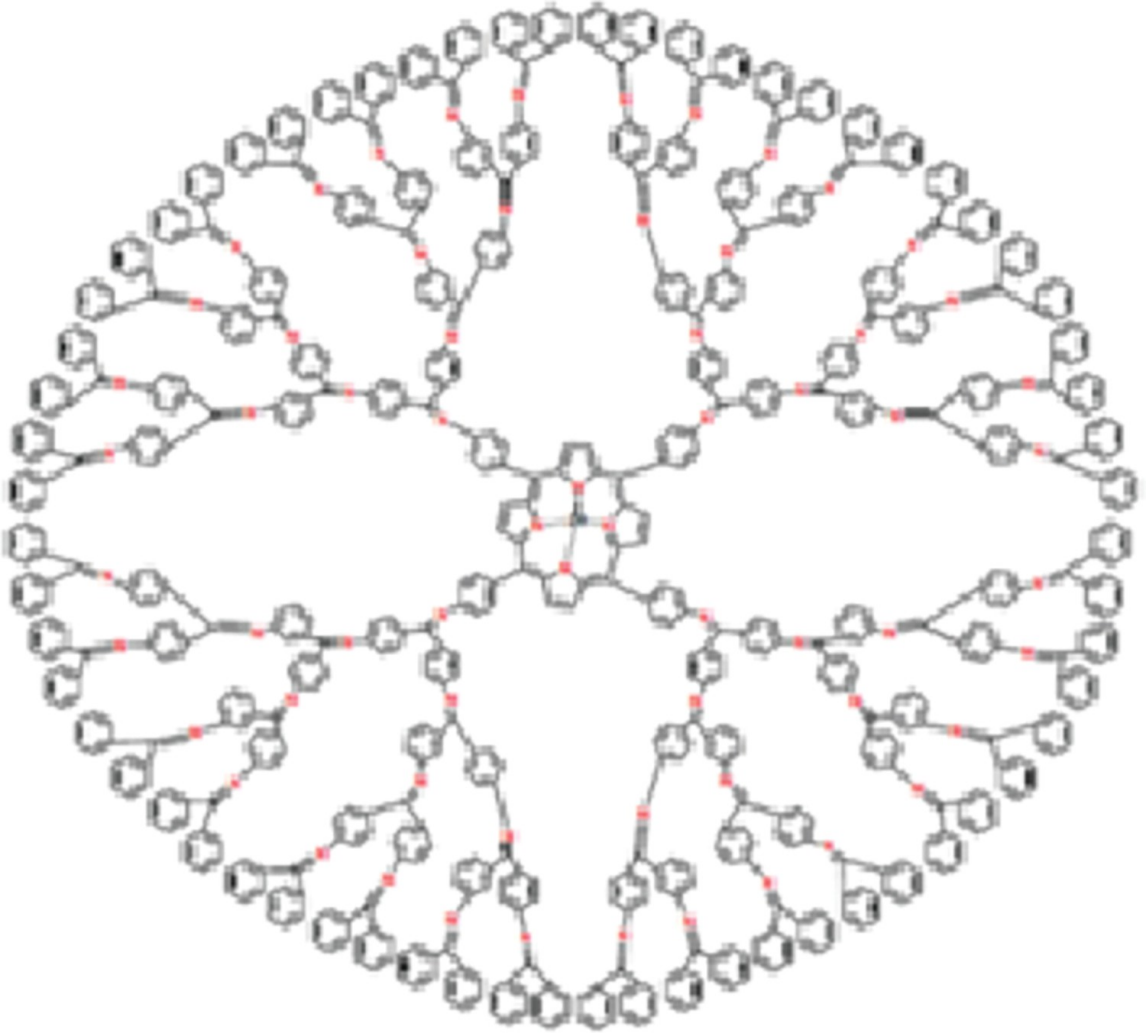
Zinc porphyrin dendrimer *DPZ*_*n*_.

In zinc porphyrin dendrimer, the central core is a porphyrin unit with zinc coordinated at its center. The dendritic branches of this dendrimer structure are created by attaching porphyrin units and other molecules to the central core in a controlled and stepwise manner. The molecular structure of zinc porphyrin dendrimer (*DPZ*_*n*_) is shown in Fig 2, where *n* is the growth stage and*n* ≥ 1.It is clear that *DPZ*_*n*_ central core is made up of 49 vertices, 24 of which are of degrees two and three, respectively, and one vertex is of degree four. Each branch of *DPZ*_*n*_ has 14(2*n* − 1) vertices, of which 11 × 2*n* − 9 vertices are of degree two and the remaining 3 × 2*n* − 5 vertices are of degree three. As a result, there are a total of 56 × 2*n* − 7 vertices in *DPZ*_*n*_. The number of edges in *DPZ*_*n*_ is 64 × 2*n*– 4. Let *E*_*ij*_ denote the number of edges of *DPZ*_*n*_ whose end vertices have degree *i* and *j* respectively. Then the edge partition of *DPZ*_*n*_ based on the degree of the end vertices of each edge is as follows: $E_{22}=16\times 2^{n}-4,E_{23}=40\times 2^{n}-16,E_{33}=8\times 2^{n}+12$, and *E*_34_ = 4. Now, using these values in the definitions of the reduced Forgotten index, the Randic index, the reduced Randic index and the Harmonic index we get the following expressions.

-
$RF({DPZ}_{n})=2^{n}(296)+110.$

-
$R({DPZ}_{n})=2^{n}(26.99659829)-3.377272109.$

-
$RR({DPZ}_{n})=2^{n}(153.9795897)+2.664570576.$

- $H({DPZ}_{n})=2^{n}(26.66666667)-3.257142857$.

### 2.3 Poly (Propyl) Ether Imine (PETIM) dendrimer

**Fig 3:**
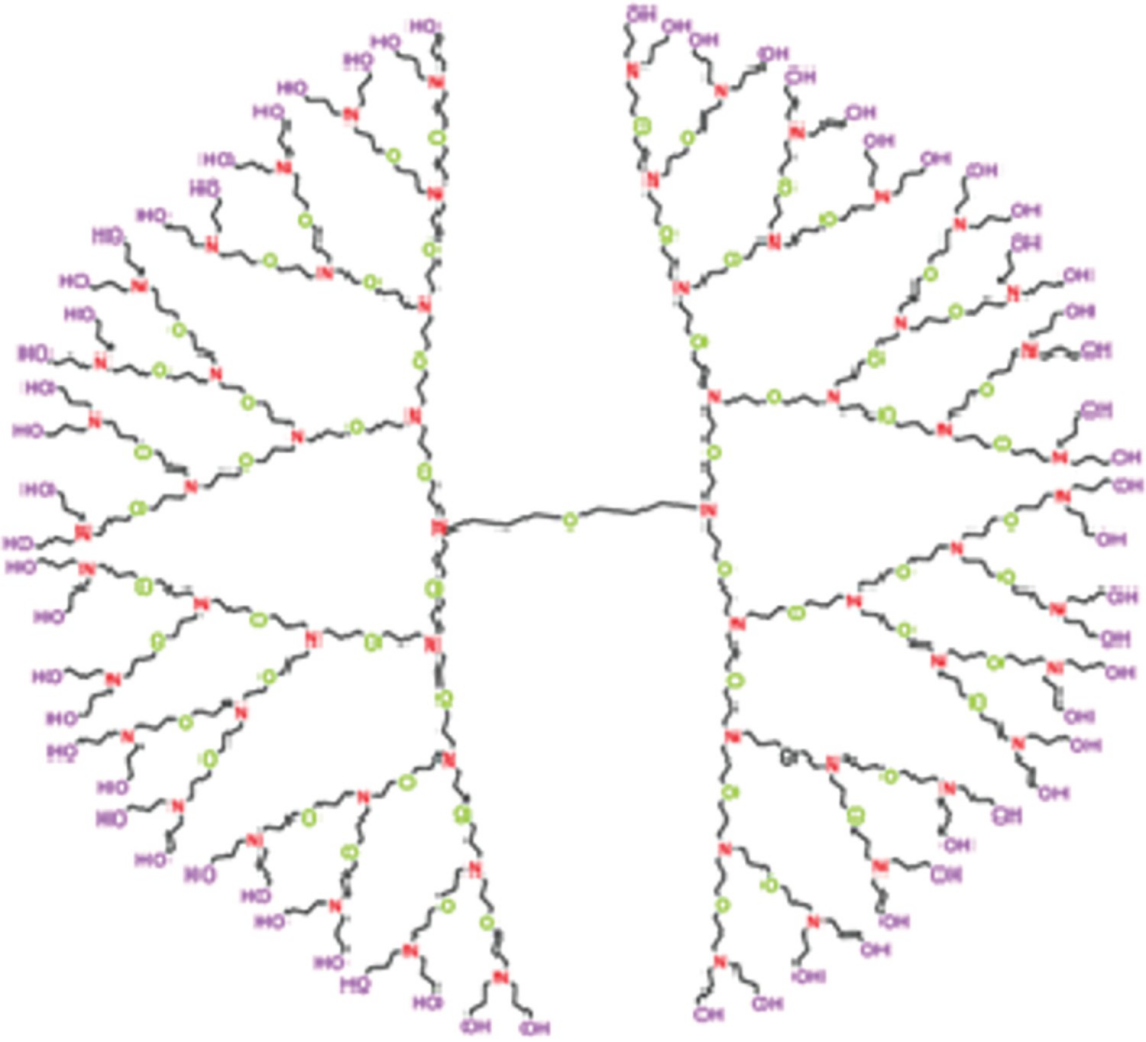
Poly (Propyl) Ether Imine (PETIM) dendrimer.

In *Poly (Propyl) Ether Imine (PETIM)* dendrimer, the branches are made up of polymer backbone composed of repeating units with propyl, ether, and imine functional group. Each generation of the *PETIM* dendrimerbegins by growing three-dimensionally from the oxygen core, branching out at each upper nitrogen atom separated by an eight-bond separator. The molecular structure of *PETIM* dendrimer is depicted in Fig 3. *PETIM* dendrimer consist of four symmetric branches and a central core having six edges. There are 6 × 2*n* − 8 edges in each branch. Therefore, in total there are 24 × 2*n* − 24 edges and 24 × 2*n*– 23 vertices in the graph of *PETIM* dendrimer. Let *E*_*ij*_ denote the number of edges of *PETIM* whose end vertices have degree *i* and *j* respectively. Then the edge partition of *PETIM* based on the degree of the end vertices of each edge is as follows: $E_{12}=2^{n+1}-4,E_{22}=16\times 2^{n}-18$, and *E*_23_ = 6×2^*n*^−6. Now, using these values in the definitions of the reduced Forgotten index, the Randic index, the reduced Randic index and the Harmonic index we get the following expressions.

- $RF(PETIM)=2^{2n}(32)-66$.
- $R(PETIM)=2^{n+1}(6.638958434)-11.44948974$.
- $RR(PETIM)=2^{n}(49.52536558)-50.69693846$.
- $H(PETIM)=2^{n}(11.73333333)-11.4$.

### 2.4 Polyamidoamine(PAMAM) dendrimer

**Fig 4:**
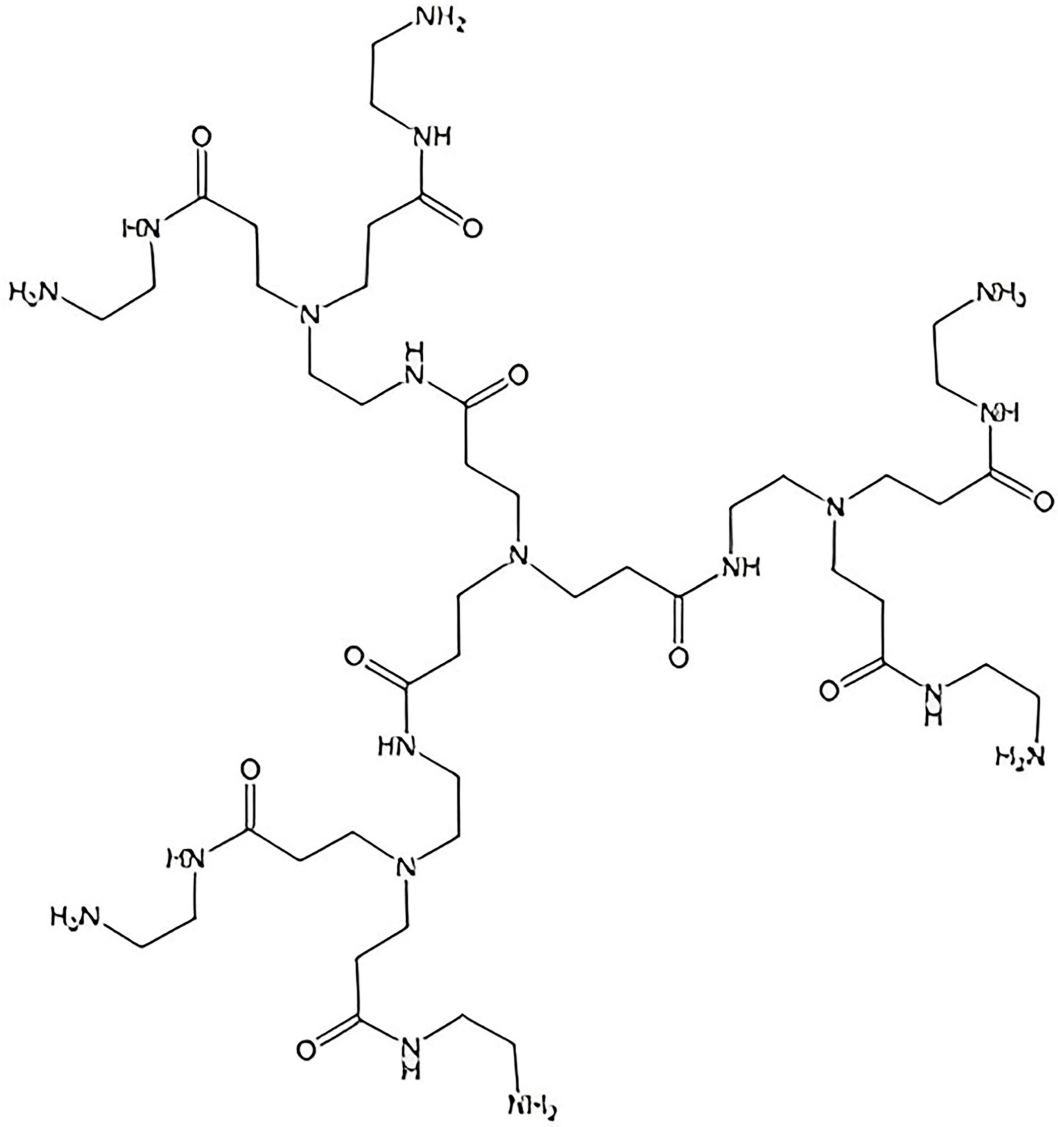
Structure of polyamidoamine (PAMAM) dendrimer.

A *Polyamidoamine(PAMAM)* dendrimer is a dendritic macromolecule where the branches are made up of polymer backbone composed of repeating units with amide and amine functional groups. The molecular structure of *PAMAM* dendrimer is depicted in Fig 4. Let *E*_*ij*_ denote the number of edges of *PAMAM* whose end vertices have degree *i* and *j* respectively. Then the edge partition of *PAMAM* based on the degree of the end vertices of each edge is as follows: $E_{12}=4\times 2^{n},E_{13}=8\times 2^{n}-4,E_{22}=24\times 2^{n}-11$, and *E*_23_ = 28×2^*n*^−14. Now, using these values in the definitions of the reduced Forgotten index, the Randic index, the reduced Randic index and the Harmonic index we get the following expressions.

- $RF(PAMAM)=(224)2^{n}-108$.
- $R(PAMAM)=(30.8781814)2^{n}-13.524877$.
- $RR(PAMAM)={(136.098973)2}^{n}-63.221059$.
- $H(PAMAM)=(29.866666)2^{n}-13.1$.

## Section 3: Chemical applicability of topological indices

Dendrimers are highly branched polymers with well-defined structures, and they can be tailored by attaching various functional groups, including benzene derivatives. Benzene derivatives are often used in dendrimer chemistry due to their versatile properties, such as aromaticity and reactivity. These derivatives can serve as building blocks for constructing dendritic branches or as functional groups that can be linked to the dendrimer surface. The inclusion of benzene derivatives can impart specific properties to dendrimers, making them suitable for various applications, including drug delivery, imaging, sensing, and catalysis.

To check the chemical applicability of the reduced forgotten index, the Randic index, the reciprocal Randic index and the Harmonic index, we have used the linear regression model to correlate our topological indices with the physicochemical properties of Benzene derivatives. We have considered eight benzene derivative namely, Benzene, Naphthalene, Phenanthrane, Anthracene, Chrysene, Benz(a)anthracene, triphenylene, and Tetracene and computed their topological indices. The value of topological indices for each Benzene derivative is shown in Table 1. The experimental values of four physical/chemical properties: boiling point, enthalpy, total *π* electron energy and molecular weight are depicted in Table 2. The value of *R*^2^ and *P* is calculated in each case (see Table 3).

**Table 1:** Values of topological indices of Benzene derivatives.

Structure name	Reduced forgotten index	Randic index	Reciprocal Randic index	Harmonic index
Benzene	12	3	12	3
Naphthalene	32	4.63299	21.797959	4.6
Phenanthrene	68	6.94949	37.696938	6.9
Anthracene	68	6.932653	37.595918	6.866667
Chrysene	96	8.932653	50.595918	8.866667
Benzo[a]anthracene	96	8.915816	50.494897	8.833333
Triphenylene	98	9.432653	52.595918	9.36667
Tetracene	96	8.898979	50.393877	8.8

**Table 2:** Physical/chemical properties of Benzene derivative.

Structure name	Boiling point	Enthalpy	*π* electron energy	Molecular weight
Benzene	78.1	80.1	75.2	8
Naphthalene	128.17	218	141.0	13.683
Phenanthrene	178.23	338	202.7	19.448
Anthracene	178.23	340	222.6	19.314
Chrysene	228.3	431	271.1	25.192
Benzo[a]anthracene	228.3	425	277.1	25.101
Triphenylene	228.3	429	275.1	25.275
Tetracene	228.3	440	310.5	25.188

**Table 3:** *R*^2^(R-squared) values to evaluate the goodness of fit.

	Reduced forgotten index	Randic index	Reciprocal Randic index	Harmonic index
Boiling point	0.9934	0.9921	0.9935	0.9914
Enthalpy	0.9798	0.9761	0.9780	0.9750
*π* electron energy	0.9668	0.9591	0.9633	0.9569
Molecular weight	0.9937	0.9931	0.9942	0.9925

From Table 3, it can be observed that the value of R-squared in each case is greater than 0.95. Also, the P value for all the cases is less than 0.0001. This suggests that the linear regression model has strong predictive power and can be relied upon for making predictions.

## Section 4: Multiple-criteria decision-making (mcdm) technique

Multi-objective decision-making (MODM) and multi-attribute decision-making (MADM) [11] are two types of MCDM. Each of these categories contains a number of approaches. Different methods utilize different normalizing procedures and treatment benefit and cost criteria differently. The Simple Additive Weighting (SAW), Technique for order preference by similarity to an ideal solution (TOPSIS) and GRA methodologies are combined in this work to create a hybrid method. SAW is particularly common in practical applications due to its simplicity. It is frequently used as a baseline against which other MCDM approaches are measured. Two distance-based techniques are TOPSIS and GRA.

In several fields, MCDM has been one of the fastest increasing problems areas [20]. Many scholars have used MCDM methodologies in the field of industrial engineering to make judgments throughout the last decade. All of the methods are capable of making decisions in the face of ambiguity, and each has its own set of benefits. Many MCDM techniques exist in literature which involve the analytical hierarchy process (AHP) [21], simple additive weighting (SAW) [22], techniques for order preference by similarity to an ideal solution (TOPSIS) [23], data envelopment analysis (DEA) [24], grey relational analysis (GRA) [25], the compromise ranking method (VIKOR) [26], the preference ranking organization method for enrichment evaluation (PROMETHEE) [27], and multi-objective optimization on the base of ratio analysis (MOORA) [28] are the most commonly used methodologies. These well-established MCDM methods employ various analysis models and decision criteria.

The Simple Additive Weighting (SAW) technique is the most basic, well-known, and widely utilized multi-criteria decision-making method. The weighted total of all attribute values determines the overall rating of a suggested solution in SAW [29]. The 3 main phases of the SAW technique are normalizing the decision matrix Y, allocating the weight matrix V, and estimating the overall rating for each choice.

A Multi-Objective Optimization Method by Ratio Analysis (MOORA), sometimes called a multicriteria or multiple attribute optimization is the process of maximizing two or more competing qualities (goals) at the same time while adhering to specified restraint. The MOORA approach, which was first described by Brauers in 2004, is a multi-objective optimization strategy that may be used to deal with a wide range of issues. In an industrial environment, it has been effectively employed to tackle a variety of complicated decision-making challenges.

TOPSIS method is commonly used to finish the decision-making process. This is owing to the concept’s simplicity, ease of comprehension, efficient computation, and ability to compare the relative performance of alternative decisions.

### 4.1 Beneficial and non-beneficial criteria and weightage

For beneficial and non-beneficial chemical properties, we looked at molecular weight, enthalpy, boiling point, and *π* electron energy of dendrimer structures. The numerical values of these properties are then correlated with the numerical values of topological indices. We have the beneficial criteria as shown in Table 4. In order to use multi-criteria decision making, we assign weights to these physical/chemical properties fulfilling the condition that sum of all weights is 1. For assigning the weight, we use entropy method.

**Table 4:** Beneficial and non beneficial criteria.

*RF*(*G*)	Enthalpy	Beneficial
***RR*(*G*)**	Boiling point	Beneficial
***H*(*G*)**	*π* electron energy	Beneficial
***R*(*G*)**	Molecular Weight	Non-Beneficial

### 4.2 Entropy method

A weighting technique that is frequently used to quantify value dispersion in decision-making is the entropy weight method (EWM). More information can be extracted and the degree of differentiation increases with increasing dispersion. In the meanwhile, the index should be given more weight, and vice versa.

Decision matrix is given below in Table 5:

**Table 5:** The decision matrix.

Alternatives	*RF*(*G*)	*RR*(*G*)	*H*(*G*)	*R*(*G*)
***D*_*n*_ *P*_*n*_**	652	231.0554837	36.51904762	39.44386315
***DPZ*_*n*_**	702	310.62375	50.07619048	50.61592446
***PAMAM***	226	151.5584309	34	35.18624182
***PETIM***	190	147.4045239	35.53333337	36.00532348

**Step 1**. Standardize the decision matrix.

To acquire the project outcomes, we normalize the decision matrix array in Table 6.


$$q_{kl}=\frac{x_{kl}}{\sum_{k=1}^{r}x_{kl}}$$

**Table 6:** Standardized decision matrix.

Alternatives	*RF*(*G*)	*RR*(*G*)	*H*(*G*)	*R*(*G*)
***D*_*n*_ *P*_*n*_**	0.368362	0.274856	0.233904	0.244611
***DPZ*_*n*_**	0.39661	0.369508	0.320737	0.313895
***PAMAM***	0.127684	0.180289	0.217769	0.223287
***PETIM***	0.107345	0.175348	0.22759	0.218207

**Step 2**.Compute the entropy value

Using the following equation, we compute the entropy measure of project outcomes in the Table 7.


$$E_{l}=-m\sum_{k=1}^{r}q_{kl}lnq_{kl}$$

In which $m=\frac{1}{\ln\left(k\right)}$

**Table 7:** Entropy values.

***E***_***l***_	**0.892325**	**0.964443**	**0.990683**	**0.991921**

**Step 3.** Identifying the target weight.

Based on the entropy notion, we define the objective weight in Table 8.


$$w_{l}=\frac{1-E_{l}}{\sum_{k=1}^{s}\left(1-E_{l}\right)}$$

**Table 8:** Target weight.

Alternatives	*RF*(*G*)	*RR*(*G*)	*H*(*G*)	*R*(*G*)
***D*_*n*_ *P*_*n*_**	0.368362	0.274856	0.233904	0.244611
***DPZ*_*n*_**	0.39661	0.369508	0.320737	0.313895
***PAMAM***	0.127684	0.180289	0.217769	0.223287
***PETIM***	0.107345	0.175348	0.22759	0.218207
***W*_*l*_**	0.670338	0.221362	0.058006	0.050293

## Section 5: Calculations of multicriteria decision making techniques (mcdm)

In an MCDM problem, all of the criteria can be divided into two groups. Benefit criteria are those that should be maximized. Cost criteria, on the other hand, are those that must be kept to a minimum. The following is an example of a common MCDM problem with *q* attributes (*A*_1_, *A*_2_,…,*A*_*q*_) and *r* criteria (*C*_1_, *C*_2_,…,*C*_*r*_)

$$Y={⦋y_{kl}⦌}_{q\times r},V={\left[v_{l}\right]}_{r}$$

Where, Y is the decision matrix, *y*_*kl*_ represent the *kth* attributes’ performance in relation to the *lth* characteristic, *V* is the weight vector, and the weightage of the *lth* set of criteria is denoted by *v*_*l*_. Because distinct standards are represented in various units of measurement, the actual decision matrix X is often beyond compare. As a result, utilizing a normalization technique, data should be turned into equivalent values. The weight vector *V* has a significant impact on alternative ranking results. The Analytic Hierarchy Process approach is frequently used to fix it. We consider dendrimer properties such as Enthalpy, molecular weight, *π* electron energy, and boiling point.

### 5.1 Saw method

The following is a summary of the entire procedure

**Step 1**. Normalize the decision matrix

Using a normalization technique, the original information should be turned into equivalent values. There have been many different normalization procedures created for the SAW method. The Max approach is the most commonly used normalizing technique. During normalization, cost criteria must be transformed into benefit criteria. The following is the normalizing procedure:

$$x_{kl}=\left\{ \begin{array}{c} x_{kl}/x_{l}^{+},\;l\in A_{max}\\ x_{l}^{-}/x_{kl},l\in A_{min} \end{array}.\right.$$

where *x*_*kl*_ represent the normalized value of the *kth* attribute to the *lth* set of criteria, $x_{l}^{+}$ is the largest number of *x*_*kl*_ in the column of *l* for advantage set of criteria, $x_{l}^{-}$ is the smallest numberof *x*_*kl*_ in the column of *l* for cost criterion, and largest and smallest are sets of advantage and cost criteria, respectively. The decision matrix is shown in Table 9.

**Table 9:** Decision matrix.

Alternatives	*RF*(*G*)	*RR*(*G*)	*H*(*G*)	*R*(*G*)
***D*_*n*_ *P*_*n*_**	652	231.0554837	36.51904762	39.44386315
***DPZ*_*n*_**	702	310.62375	50.07619048	50.61592446
***PAMAM***	226	151.5584309	34	35.18624182
***PETIM***	190	147.4045239	35.53333337	36.00532348
**Best**	702	310.6238	50.07619	35.18624

Now, we normalize the decision matrix, see Table 10.

**Table 10:** Normalized decision matrix.

Alternatives	*RF*(*G*)	*RR*(*G*)	*H*(*G*)	*R*(*G*)
***D*_*n*_ *P*_*n*_**	0.928775	0.743844	0.72927	0.892059
***DPZ*_*n*_**	1	1	1	0.695161
***PAMAM***	0.321937	0.487916	0.678965	1
***PETIM***	0.270655	0.474544	0.709585	0.977251

**Step 2.** Assign weight *V* to each criterion

$$V=[v_{1},v_{2},\ldots ,v_{r}].$$

In Table 11, we have allocated the weights to chemical/physical properties.

**Table 11:** Weights of chemical/physical properties.

Alternatives	*RF*(*G*)	*RR*(*G*)	*H*(*G*)	*R*(*G*)
***D*_*n*_ *P*_*n*_**	0.9287749288	0.6379615906	0.7292696843	0.8920587136
***DPZ*_*n*_**	1	0.4745436365	1	0.6951614970
***PAMAM***	0.3219373219	0.9725920427	0.6789653860	1
***PETIM***	0.2706552707	1	0.7095853944	0.9772510956
***weightage***	0.25	0.25	0.25	0.25

**Step 3**. Calculating the ranking score:

$$A_{k}=\sum_{l=1}^{r}v_{l}\cdot x_{kl}.$$

Where *A*_*k*_ is the *kth* alternative’s ranking score, *v*_*l*_ is the *lth* criterion’s weight, and *x*_*kl*_ is the *kth* alternativesnormalized performance against the *lth* criterion.

We have calculated the ranking in Table 12 and can see that *DPZ*_*n*_ is ranked first.

**Table 12:** Ranking of dendrimer structures.

Alternatives	*RF*(*G*)	*RR*(*G*)	*H*(*G*)	*R*(*G*)	Ai	Rank
***D*_*n*_ *P*_*n*_**	0.232194	0.185961	0.182317	0.223015	0.823487	2
***DPZ*_*n*_**	0.25	0.25	0.25	0.17379	0.92379	1
***PAMAM***	0.080484	0.121979	0.169741	0.25	0.622205	3
***PETIM***	0.067664	0.118636	0.177396	0.244313	0.608009	4

### 5.2. MOORA method

The MOORA method’s steps are

**STEP 1**. Create a decision matrix

At first, we create a decision matrix *y*_*kl*_ in shown in Table 13.


$$y_{kl}=\left[\begin{array}{cccc} y_{11} & y_{12} & . & y_{1r}\\ y_{21} & y_{22} & . & y_{2r}\\ . & . & . & .\\ y_{q1} & y_{q2} & & y_{qr} \end{array}\right].$$

**Table 13:** Decision matrix.

Alternatives	RF(G)	H(G)	RR(G)	R(G)
***D*_*n*_ *P*_*n*_**	652	36.51904762	231.0554837	39.44386315
***DPZ*_*n*_**	702	50.07619048	310.62375	50.61592446
***PAMAM***	226	34	151.5584309	35.18624182
***PETIM***	190	35.53333337	147.4045239	36.00532348
**Best**	702	50.07619048	310.62375	35.18624182
***weightage***	0.25	0.25	0.25	0.25

**Step 2.**Normalize the Decision Matrix

$$x_{kl}^{*}=x_{kl}/\sqrt{\left[\sum_{k=1}^{q}x_{kl}^{2}\right]}(l=1,2,3,\ldots ,r).$$

Using the above equation, we normalize the decision matrix, see Table 14.

**Table 14:** Normalized decision matrix.

Alternatives	RF(G)	H(G)	RR(G)	R(G)
***D*_*n*_ *P*_*n*_**	0.650348909	0.461568666	0.523813069	0.483610056
***DPZ*_*n*_**	0.700222292	0.632919037	0.704197871	0.620587542
***PAMAM***	0.225427689	0.429730118	0.34358971	0.431408564
***PETIM***	0.189518854	0.449110104	0.334172618	0.441451093

**Step 3.** Optimize attributes.

These normalized performances are added in case of maximization (for favorable qualities) and lowered in case of minimization in multi-objective optimization (for non-beneficial attributes). The problem of optimization therefore becomes:

$$a_{k}=\sum_{l=1}^{d}x_{kl}^{*}-\sum_{l=d+1}^{r}x_{kl}^{*}.$$

Where g is the number of attributes to be maximized, (n-g) denotes the number of attributes to be minimized, and *a*_*i*_ denotes the alternative value’s normalized value against all attributes. It is common to notice that some characteristics are more significant than others in various situations. The attribute can be multiplied by the corresponding weight to give more importance to the attribute. When the weight of this attribute is considered,

$$a_{k}=\sum_{l=1}^{d}v_{l}x_{kl}^{*}-\sum_{l=d+1}^{r}{v_{l}x}_{kl}^{*}(l=1,2,3,\ldots ,r).$$

Depending on the maximal number (beneficial attribute) and minimal number (unbeneficial attribute) in the choice matrix, the value of *a*_*i*_ can be positive or negative.

The ordinal rank of *a*_*i*_ indicates the final choice. As a result, the best choice has the greatest *a*_*i*_ value, but the worst choice has the least. $v_{l}x_{kl}^{*}$ is calculated in Table 15.

**Table 15:** The values of $v_{l}x_{kl}^{*}$.

Alternatives	RF(G)	H(G)	RR(G)	R(G)
***D*_*n*_ *P*_*n*_**	0.162587227	0.115392	0.130953	0.120902514
***DPZ*_*n*_**	0.175055573	0.15823	0.176049	0.155146885
*PAMAM*	0.056356922	0.107433	0.085897	0.107852141
***PETIM***	0.047379713	0.112278	0.083543	0.110362773

Next, we calculate *A*_*k*_ and the ranking score in shown in Table 16.

**Table 16:** Calculating *A*_*k*_ and ranking.

Alternatives	*A*_*k*_	Rank
***D*_*n*_ *P*_*n*_**	0.28803	2
***DPZ*_*n*_**	0.354188	1
***PAMAM***	0.141835	3
***PETIM***	0.132838	4

### 5.3. TOPSIS method

The steps in TOPSIS method are

**Step 1.** Normalize the decision matrix

$${\bar{R}}_{kl}=R_{kl}/\sqrt{\left[\sum_{l=1}^{r}R_{kl}^{2}\right]}.$$

Here ${\bar{R}}_{kl}$ is the normalized matrix see Table 17.

**Table 17:** Normalized matrix.

Alternatives	RF(G)	H(G)	RR(G)	R(G)
***D*_*n*_ *P*_*n*_**	0.6503489089	0.4615686659	0.523813069	0.483610056
***DPZ*_*n*_**	0.7002222915	0.6329190365	0.704197871	0.620587542
***PAMAM***	0.2254276893	0.4297301179	0.34358971	0.431408564
***PETIM***	0.1895188538	0.4491101041	0.334172618	0.441451093

**Step 2.** Calculate weights of normalized matrix, see Table 18.


$$V_{kl}=\bar{R_{kl}}W_{l}$$

**Table 18:** Weights of normalized matrix.

Alternatives	*RF*(*G*)	*H*(*G*)	*RR*(*G*)	*R*(*G*)
***D*_*n*_ *P*_*n*_**	0.162587227	0.115392	0.130953	0.120902514
***DPZ*_*n*_**	0.175055573	0.15823	0.176049	0.155146885
***PAMAM***	0.056356922	0.107433	0.085897	0.107852141
***PETIM***	0.047379713	0.112278	0.083543	0.110362773

**Step 3.**Calculate ideal best and worst value.

Using the above criteria, we determined ideal best and worst in Table 19.

**Table 19:** Ideal best and worst values.

Alternatives	*RF*(*G*)	*H*(*G*)	*RR*(*G*)	*R*(*G*)
Best	0.175056	0.15823	0.176049	0.107852
Worst	0.04738	0.107433	0.083543	0.155147

**Step 4.**Calculate Euclidean distance from the ideal best

$$T_{k}^{+}=\sqrt{\sum_{l=1}^{q}{\left(V_{kl}-V_{l}^{+}\right)}^{2}}.$$

**Step 5.** Calculate Euclidean distance from the ideal worst

$$T_{k}^{-}=\sqrt{\sum_{l=1}^{q}{\left(V_{kl}-V_{l}^{-}\right)}^{2}}.$$

**Step 6.**Calculate performance score

$$S_{k}=\frac{T_{k}^{-}}{T_{k}^{+}+T_{k}^{-}}.$$

After calculating the performance score, Rank the best alternatives according to *S*_*k*_ in descending order. Table 20 shows the performance score and ranking. Here *DPZ*_*n*_ is on rank 1 and the ranking of remaining is as follow *D*_*n*_*P*_*n*_>*PAMAM*>*PETIM*.

**Table 20:** Ranking of dendrimers.

Alternatives	*RF*(*G*)	*H*(*G*)	*RR*(*G*)	*R*(*G*)	$T_{k}^{+}$	$T_{k}^{-}$	*S*_*k*_	RANK
***D*_*n*_ *P*_*n*_**	0.16258	0.11539	0.13095	0.12090	0.06476	0.12944	0.66652	2
***DPZ*_*n*_**	0.17505	0.15823	0.17604	0.15514	0.04729	0.16564	0.77789	1
***PAMAM***	0.05635	0.10743	0.08589	0.10785	0.15747	0.04819	0.23434	3
***PETIM***	0.04738	0.11227	0.08354	0.11036	0.16424	0.04504	0.21522	4

## Section 6: Graphical representation

**Fig 5:**
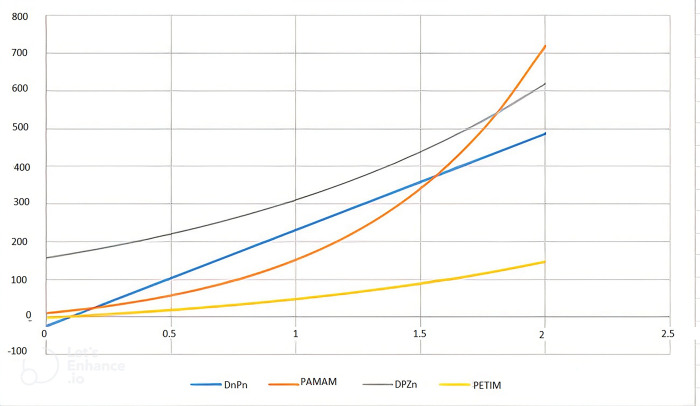
2D representation of reciprocal randic index of dendrimer structure.

**Fig 6:**
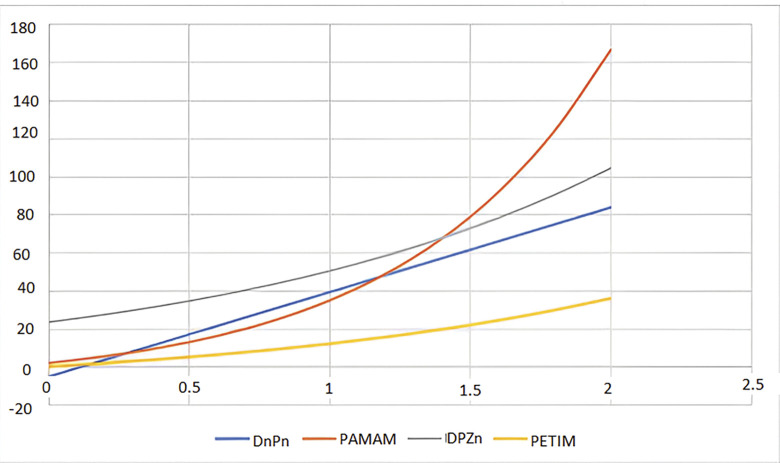
2D representation of randic index of dendrimer structure.

**Fig 7:**
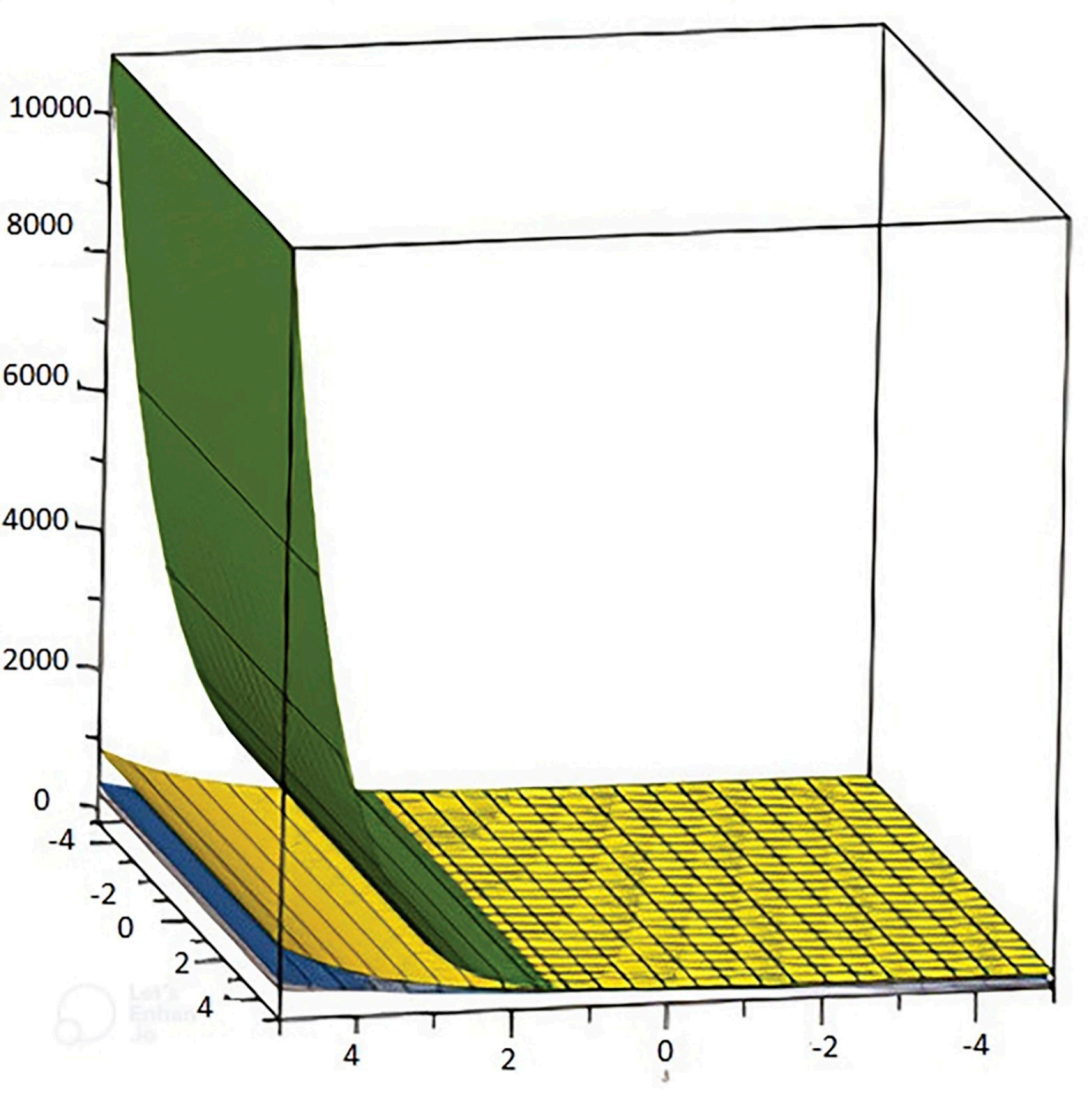
3D reflection of the harmonic index of dendrimer structure.

**Fig 8:**
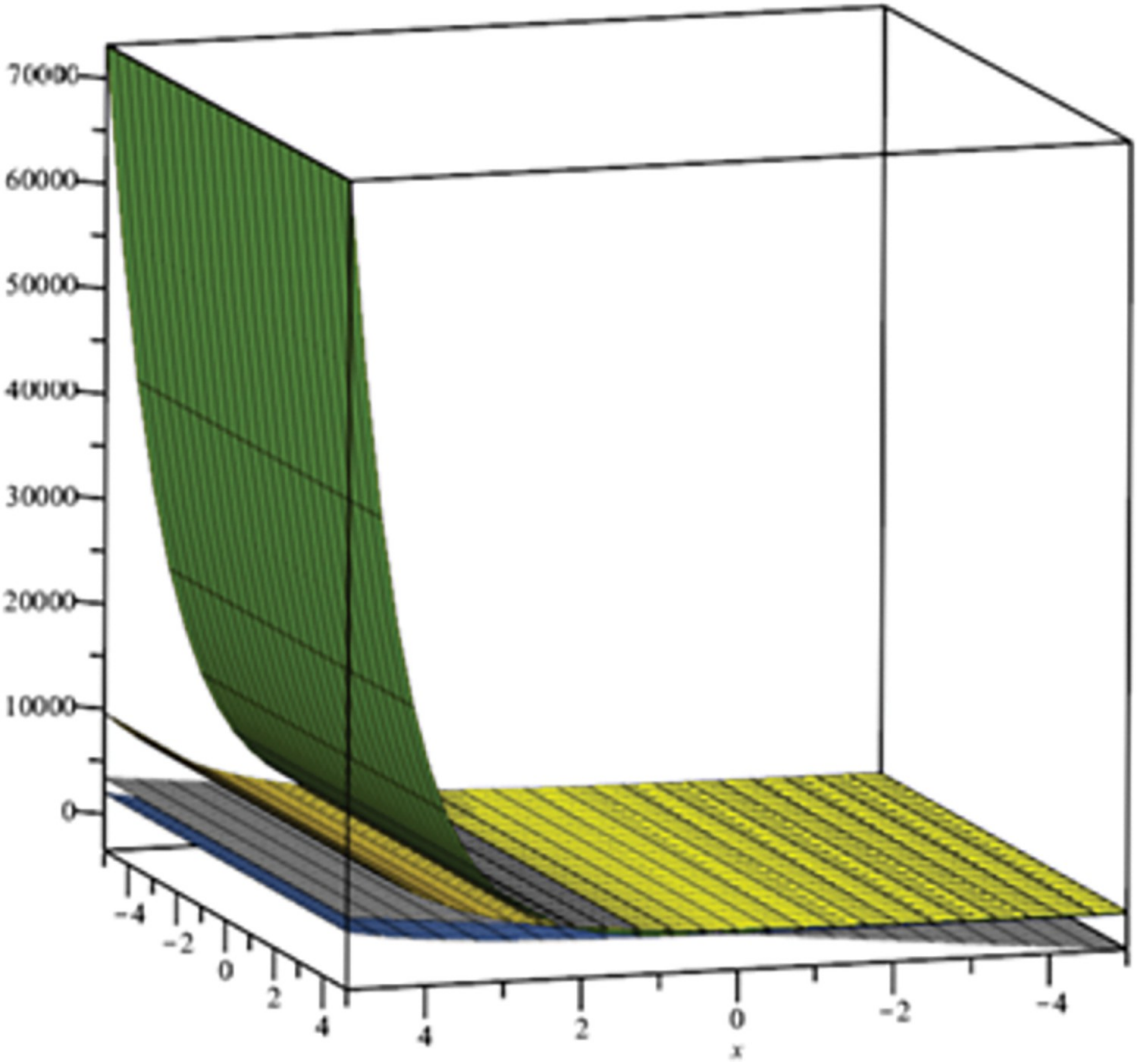
3D reflection of the reduced forgotten topological index of dendrimer structure.

We can see the 2 Dimensional graphical representation of the randic index in Fig 5 and the reciprocal randic index in Fig 6. In Fig 7 we can see the 3D graphs of the harmonic index and in Fig 8we can see the 3D graph of reduced forgotten topological index where the colour representation is as follows: grey, gold, green, and Niagara Azure represent *D*_*n*_*P*_*n*_, *DPZ*_*n*_, *PAMAM*, and *PETIM* dendrimer respectively. In the graphs, the values of n are along the x-axis, while the topological indices are on the y-axis.

## Section 7: Conclusion

We have determined degree-based topological indices of some classes of dendrimer structures. We applied multi-criteria decision-making techniques to determine which dendrimer is superior in terms of chemical characteristics. MCDM offers a framework for choosing, classifying, and prioritising resources as well as assistance with the overall assessment.

Although studies have demonstrated that several dendrimers have their own therapeutic uses, largely because of their antifungal, antibacterial, and cytotoxic qualities, dendrimers are also valuable as transport or carrier systems for medications and genes. Another application for dendrimers is as a solubilizer. We have observed that Zinc Porphyrin dendrimer is better than others using TOPSIS, SAW, and MOORA techniques, followed by Porphyrine dendrimer, PAMAM dendrimer, and finally PETIM dendrimer. Since Zinc Porphyrine is superior than other dendrimers, we can employ it as a transport or carrier system for drugs and genes. If we need to create a pharmaceutical or drug delivery agent and have a range of dendrimers accessible, we may utilise these decision-making techniques to decide which structure is best for our purposes in chemistry.
